# Superinfection by Trichomonas, a Second Reason to Add Metronidazole to COVID-19 Treatment; a Letter to Editor

**Published:** 2020-10-22

**Authors:** Christophe Duboucher

**Affiliations:** 1 Laboratory of Pathology, C.H.I. de Poissy / Saint-Germain, Saint-Germain-en-Laye, France.

**Keywords:** COVID-19, metronidazole, respiratory distress syndrome, adult, SARS-CoV-2, trichomonas infections

**Dear Editor,**

All countries are struggling against the COVID-19 pandemic. One million deaths have been recorded. Acute respiratory distress syndrome (ARDS) is the major complication. No antiviral therapy has been shown to be clearly effective for reducing the rate of mortality in published randomised controlled trials.

In an article published in the last issue of Archives of Academic Emergency Medicine, metronidazole is suggested to be tested in clinical trials (1). *In vitro* and *in vivo* studies have revealed that metronidazole could decrease the levels of several cytokines. It could also decrease neutrophil-generated reactive oxygen species, and thus could counteract majority of the immunopathological manifestations of the COVID-19 infection.

Metronidazole could be included in clinical trials for another reason. More than ten years ago we observed that lungs of patients diseased from ARDS, show superimposed infection by trichomonads (2).

In this situation, local hypoxic conditions could be the main factor favoring trichomonad infection development as alveolar lumens are obliterated by fibrin and cellular debris.

If our observations show that trichomonads infection could develop during the late phase of ARDS, the deleterious action of trichomonads remains to be proven. Nevertheless, amoeboid transformation argues for aggressiveness of trichomonads (3).

When facing the current COVID-19 pandemic, the major medical complication of which is ARDS, the presence of trichomonads should not be overlooked or considered an anecdotal event. It seems fair to assess the potential deleterious role of trichomonads in prospective clinical trials.

The same observation of superimposed infection by trichomonads has been made during the course of *Pneumocystis jirovecii* pneumonia (PJP) (4).

However, since our publication in 2005, observations of trichomonads in the course of PJP and of ARDS have not been made or commented on by other cytopathologists or parasitologists. Readers who are not observers may have doubts, but observers may see these unidentified cells in bronchoalveolar lavage fluids (BALF), and worry about their nature.

Nevertheless, there is an explanation for this occultation. In alveolar lumens, when adhering to epithelial cells of host, trichomonads evolve from a flagellated form to an amoeboid form. When transforming into amoeboid form, they develop pseudopods and lose their flagella. Thus, they look like anonymous cells that have lost their distinctive marks and can mimic human cells. So, on slides colored using MGG, amoeboid trichomonads do not harbor a familiar appearance neither for parasitologists nor for cytopathologists. 

It is difficult to find reports in the literature before 2005, when the word « trichomonad » was not used as keyword of indexation. The unique article we found, in which these « alien cells » are presented, was published in 2001 in Acta Cytologica by Jan Jacobs et al. (5). The authors described « non-identified cells » almost exclusively in BALF from human immunodeficiency virus (HIV)-infected patients. Unfortunately, the authors failed to ask themselves the following question: « are these cells human cells? ».

Parasitologists and cyto-pathologists need to be convinced by pictures. Microphotographs from different cases of ARDS and PJP are visible at https://www.trichomoniasis-pathology.org/
